# Effectiveness and safety of tenofovir amibufenamide and its comparison with tenofovir alafenamide in patients with chronic hepatitis B: results from a retrospective real-world study

**DOI:** 10.3389/fphar.2023.1165990

**Published:** 2023-06-01

**Authors:** Lanqing Li, Jing Zhou, Yujing Li, Fada Wang, Dongmei Zhang, Menglan Wang, Yachao Tao, Enqiang Chen

**Affiliations:** Center of Infectious Diseases, West China Hospital of Sichuan University, Chengdu, China

**Keywords:** chronic hepatitis B, tenofovir amibufenamide, virological response, renal dysfunction, lipid profiles

## Abstract

**Background/aim:** Tenofovir amibufenamide (TMF) has shown potent antiviral efficacy in randomized clinical studies. This study aimed to reveal the effectiveness and safety of tenofovir amibufenamide in the real world and compared tenofovir amibufenamide to tenofovir alafenamide (TAF) in patients with chronic hepatitis B (CHB).

**Methods:** In this retrospective study, tenofovir amibufenamide-treated chronic hepatitis B patients were divided into treatment-naive (TN) and treatment-experienced (TE) groups. Furthermore, tenofovir alafenamide-treated patients were enrolled using the propensity score matching method (PSM). We assessed the virological response (VR, HBV DNA < 100 IU/mL) rate, renal function, and blood lipid changes during 24 weeks of treatment.

**Results:** Virologic response rates at week 24 were 93% (50/54) in the treatment-naive group and 95% (61/64) in the treatment-experienced group. The ratios of alanine transaminase (ALT) normalization were 89% (25/28) in the treatment-naive group and 71% (10/14) in the treatment-experienced group (*p* = 0.306). Additionally, serum creatinine decreased in both the treatment-naive and treatment-experienced groups, (−4.44 ± 13.55 μmol/L *vs.* −4.14 ± 9.33 μmol/L, *p* = 0.886), estimated glomerular filtration rate (eGFR) increased (7.01 ± 12.49 ml/min/1.73 m^2^ *vs.* 5.50 ± 8.16 ml/min/1.73 m^2^, *p* = 0.430), and low-density lipoprotein cholesterol (LDL-C) levels increased (0.09 ± 0.71 mmol/L *vs.* 0.27 ± 0.68 mmol/L, *p* = 0.152), whereas total cholesterol/high-density lipoprotein cholesterol (TC/HDL-C) levels decreased continuously from 3.26 ± 1.05 to 2.49 ± 0.72 in the treatment-naive group and from 3.31 ± 0.99 to 2.88 ± 0.77 in the treatment-experienced group. Using propensity score matching, we further compared virologic response rates between the tenofovir amibufenamide and tenofovir alafenamide cohorts. Virologic response rates in treatment-naive patients were higher in the tenofovir amibufenamide cohort [92% (35/38) *vs.* 74% (28/38), *p* = 0.033]. Virologic response rates in treatment-experienced patients showed no statistical difference between the tenofovir amibufenamide and tenofovir alafenamide cohorts.

**Conclusion:** Tenofovir amibufenamide had profound antiviral effectiveness and no adverse effects on renal function or blood lipids. Additionally, tenofovir amibufenamide was more efficient than tenofovir alafenamide in inhibiting viral replication, which needs to be demonstrated in future studies.

## 1 Introduction

Chronic hepatitis B (CHB), caused by the hepatitis B virus (HBV), is a worldwide infectious disease that can progress to liver fibrosis, cirrhosis, hepatocellular carcinoma, and even liver-related death without timely and effective treatment. In 2019, it was estimated that approximately 316 million people had CHB worldwide, and HBV-related disease resulted in approximately 0.06 million deaths worldwide (GBD, 2019 Hepatitis B Collaborators, 2022). Antiviral therapy is indispensable for chronic active hepatitis B because it can help reduce the risk of liver-related complications.

Currently, antiviral therapies available for CHB are divided into two categories: pegylated interferon-α agents and nucleoside/nucleotide analogs. Pegylated interferon-α is regarded as a first-line drug that can achieve HBV DNA reduction through immunologic control. Although there are more chances to achieve HBeAg and HBsAg seroclearance, the HBV DNA undetectable rate with pegylated interferon-α was approximately 30%, even during 3 years of follow-up (Sonneveld and Janssen, 2011). Entecavir (ETV), tenofovir (TDF), and tenofovir alafenamide (TAF) are also recommended as first-line antiviral regimens owing to their potent antiviral efficacy and high barrier to HBV resistance (Yim et al., 2020). A 10-year study of TDF showed that approximately 99% of CHB patients maintained virologic response (HBV DNA <29 IU/mL), but TDF was at a disadvantage due to safety concerns, which manifested as renal impairment, bone mineral density decrease, bone fractures, and so on (Marcellin et al., 2019). Although TAF could be an alternative for CHB patients with renal and bone abnormalities, the lipid-increasing effect was still an issue requiring attention in clinical practice. ETV had fewer adverse effects, and the 5-year probability of drug resistance was approximately 1.2% (Scott and Keating, 2009), although its viral suppression ability was inferior to that of TDF. In addition, some studies revealed that patients treated with TDF were at a lower risk of HCC when compared with ETV (Choi et al., 2021). Current anti-HBV drugs have their own advantages and disadvantages, and safer drugs with highly potent antiviral efficacy are still needed.

Tenofovir amibufenamide (TMF) is a novel prodrug of tenofovir. The structure of TMF is similar to that of tenofovir alafenamide (TAF) except for an additional methyl group, resulting in better liposolubility and cell membrane penetration and eventually slightly better activity than TAF *in vitro* (Zhang et al., 2021). TMF shares a similar metabolic pathway with TAF, which is ultimately transformed to TFV via carboxylesterase and cathepsin A, which are predominantly expressed in HBV-infected hepatocytes and play a role in the intracellular activation of TMF (Agarwal et al., 2015; Zhang et al., 2021). A recent 48-week randomized clinical trial demonstrated that TMF is a better choice for the treatment of CHB than TDF because of its non-inferior efficacy and better safety profile. A previous study showed that the therapeutic effect of TAF was similar to that of TDF, but with improved renal and bone safety (Liu et al., 2021). However, the ability of TAF to inhibit virus replication in untreated CHB patients is not better (Chen et al., 2021; Farag et al., 2021). TMF was approved by the National Medical Products Administration (NMPA) and was launched in China in June 2021. To date, no real-world research articles highlighting TMF have been published. The present study aimed to report the short‐term effectiveness and safety of TMF during 24 weeks of treatment and its comparison with TAF in patients with CHB.

## 2 Materials and methods

### 2.1 Study design and patients

This was a retrospective study conducted in the outpatient clinic of West China Hospital between July 2021 and April 2022. Patients who met the diagnostic and antiviral treatment criteria of the APASL guidelines were included (Sarin et al., 2016). The inclusion criteria were as follows: patients who were chronically infected with HBV and HBsAg-positive for at least 6 months and underwent treatment for no less than 24 weeks if received anti-HBV treatment before enrollment. The patients could be classified into treatment-naive or treatment-experienced groups. Exclusion criteria were as follows: 1) co-infection with hepatitis C, hepatitis D, and HIV; 2) patients with serum creatinine less than 50 ml/min/1.73 m^2^; 3) patients treated with interferon or under combined treatment with other anti-HBV drugs; 4) patients receiving radiotherapy, chemotherapy, and immunosuppressive therapy due to cancer or other severe diseases; 5) patients with decompensated cirrhosis or hepatocellular carcinoma; and 6) patients with incomplete data. All the enrolled patients received 25 mg of TMF once daily orally and were divided into the treatment-naive (TN) and treatment-experienced (TE) groups. Furthermore, a cohort of 161 patients treated with TAF was previously assessed, which included 49 TN patients and 112 TE patients. The propensity score matching method (PSM) was used to reduce heterogeneity between the TMF and TAF cohorts, with respect to age, gender, HBeAg status, baseline HBV DNA, and ALT levels.

This study strictly adhered to the ethical guidelines of the 1975 Declaration of Helsinki and was approved by the Ethics Committee of West China Hospital of Sichuan University (serial number, 2022-11-30). Furthermore, the study was registered in the Chinese Clinical Trial Registry (ChiCTR2300070261).

### 2.2 Endpoints

The primary effectiveness endpoint was the virologic response (VR), which was defined as the serum HBV DNA level <100 IU/mL at week 24. Secondary effectiveness endpoints were defined as the ratio of normal ALT (ALT ≤40 U/L), HBeAg loss, and qHBsAg levels from the baseline to week 24. Safety endpoints were renal function and blood lipids as measured by serum creatinine, estimated glomerular filtration rate (eGFR), serum phosphorus, LDL-C, and TC/HDL.

### 2.3 Statistical analysis

Continuous variables with normal distribution were expressed as mean ± standard deviation (SD) and compared with *t*-tests. Categorical variables were expressed as numbers (percentages) and compared using a chi-squared or Fisher’s exact test. A *p*-value <0.05 was considered significant for all statistical tests. All statistical analyses were performed using IBM SPSS software version 25.0.

## 3 Results

### 3.1 Baseline characteristics

**FIGURE 1:**
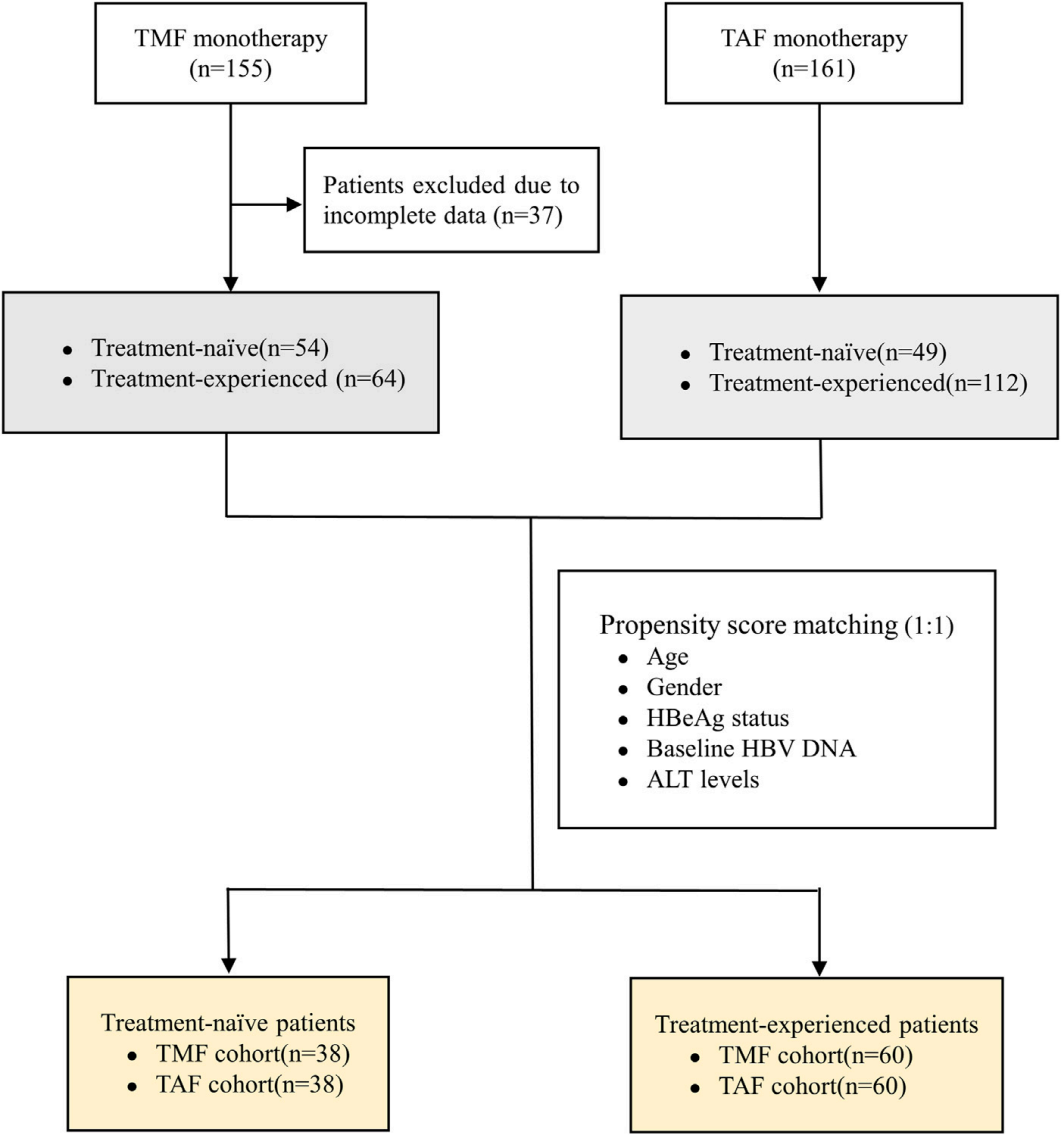
Flowchart of the study population.

As shown in Figure 1, 155 patients were enrolled, of which 118 patients who completed 24 weeks of treatment were finally included in the analysis (54 patients in the TN group and 64 patients in the TE group). The baseline characteristics of enrolled patients are described in Table 1. In the TN group, the mean age was 42.17 ± 10.79 years, 54% (29/54) of the patients were male, and 50% (27/54) of the patients were HBeAg-positive; their serum qHBsAg level was 3.58 ± 0.73 log10 IU/ml, and the baseline HBV DNA level was 5.16 ± 1.73 log10 IU/mL; the baseline ALT level was 43.83 ± 22.84 U/L, and the normal ALT ratio was 48% (26/54). In the TE group, the mean age was 37.19 ± 7.52 years, 72% (46/64) of the patients were male, and 41% (26/64) of the patients were HBeAg-positive; the serum qHBsAg level was 2.85 ± 0.82 log10 IU/ml, and 97% (62/64) of the patients had undetectable HBV DNA; the baseline ALT level was 34.37 ± 26.80 U/L, and the normal ALT ratio was 78% (50/64). There were no differences between the TN and TE groups in terms of the HBeAg status, eGFR, serum creatinine, serum phosphorus levels, TC, LDL-C, HDL-C, and TC/HDL-C.

**TABLE 1:** Baseline clinical characteristics of enrolled patients.

	Total (*n* = 118)	TN (*n* = 54)	TE (*n* = 64)	*p*-value
Age (years)	39.47 ± 9.45	42.17 ± 10.79	37.19 ± 7.52	0.005
Gender, *n* (%)				
Male patients	75 (64)	29 (54)	46 (72)	0.041
Female patients	43 (36)	25 (46)	18 (28)	
HBeAg, *n* (%)				
Positive	53 (45)	27 (50)	26 (41)	0.308
Negative	65 (55)	27 (50)	38 (59)	
HBsAg, log10 IU/ml	3.18 ± 0.86	3.58 ± 0.73	2.85 ± 0.82	0.000
HBV DNA, log10 IU/ml	5.15 ± 1.71	5.16 ± 1.73	4.96 ± 1.18	NA
≤2, *n* (%)	62 (53)	0	62 (97)	Not performed
>2, *n* (%)	56 (47)	54 (100)	2 (3)	
PLT, ×10^9^/L	176.08 ± 52.77	173.80 ± 51.57	178.02 ± 54.09	0.667
TBI, mmol/L	15.22 ± 6.14	16.58 ± 6.53	14.07 ± 5.59	0.027
ALB, g/L	45.09 ± 5.07	43.94 ± 4.71	46.05 ± 5.20	0.024
ALT, U/L	38.70 ± 25.41	43.83 ± 22.84	34.37 ± 26.80	0.043
≤40, *n* (%)	76 (64)	26 (48)	50 (78)	0.016
>40, *n* (%)	42 (36)	28 (52)	14 (22)	
AST, U/L	30.70 ± 16.93	34.06 ± 16.54	27.88 ± 16.87	0.048
Scr, μmol/L	71.77 ± 15.34	70.15 ± 15.48	73.14 ± 15.21	0.293
eGFR, mL/min/1.73 m^2^	108.18 ± 14.10	109.70 ± 15.57	106.89 ± 12.72	0.284
<60, *n* (%)	0	0 (0)	0 (0)	NA
<90, *n* (%)	16 (14)	7 (13)	9 (14)	
≥90, *n* (%)	102 (86)	47 (87)	55 (86)	
Serum phosphorus, mmol/L	1.11 ± 0.17	1.14 ± 0.14	1.08 ± 0.19	0.093
TC, mmol/L	4.13 ± 0.97	4.12 ± 0.95	4.14 ± 0.99	0.922
HDL-C, mmol/L	1.31 ± 0.27	1.31 ± 0.24	1.30 ± 0.31	0.901
LDL-C, mmol/L	2.99 ± 0.10	3.15 ± 0.93	2.85 ± 1.04	0.104
TC/HDL	3.29 ± 1.01	3.26 ± 1.05	3.31 ± 0.99	0.814

PLT, platelet; TBIL, total bilirubin; ALB, albumin; AST, aspartate aminotransferase; ALT, alanine aminotransferase; Scr, serum creatinine; eGFR, estimated glomerular filtration rate; TC, total cholesterol; HDL-C, high-density lipoprotein cholesterol; LDL-C, low-density lipoprotein cholesterol; NA, not available.

After PSM, 38 pairs of TN patients and 60 pairs of TE patients were generated between the TMF and TAF cohorts. In TN patients, the mean serum HBV DNA level was higher in the TMF cohort (5.42 ± 1.72 log10 IU/mL *vs.* 4.57 ± 1.93 log10 IU/mL, *p* = 0.047). In TE patients, there were statistical differences in HBeAg ratios with more HBeAg-positive patients in the TAF cohort (65% *vs.* 42%, *p* = 0.010). Details of baseline characteristics after PSM between patients treated with TMF and TAF are listed in Table 2.

**TABLE 2:** Baseline of patients treated with TMF and TAF after PSM.

	TN		*p*	TE		*p*
	TMF (*n* = 38)	TAF (*n* = 38)		TMF (*n* = 60)	TAF (*n* = 60)	
Age, years	42.16 ± 9.79	40.79 ± 10.68	0.562	37.62 ± 7.42	35.23 ± 6.87	0.070
Male patients, *n* (%)	21 (55)	22 (58)	0.817	43 (72)	44 (73)	0.838
HBeAg-positive, n/N (%)	24 (63)	23 (61)	0.813	25 (42)	39 (65)	0.010
HBsAg, log10 IU/mL	3.70 ± 0.70	3.46 ± 0.68	0.125	2.87 ± 0.70	2.99 ± 0.88	0.248
HBV DNA, log10 IU/ml	5.42 ± 1.72	4.57 ± 1.93	0.047	Undetectable	3.31 ± 0.33	NA
≤2, *n* (%)	0.00	0.00	NA	60	58	NA
>2, *n* (%)	38 (100)	38 (100)		0	2	
ALT, U/L	46.79 ± 22.97	39.34 ± 27.65	0.206	34.29 ± 27.31	29.01 ± 18.46	0.217
Ratio of normal ALT	17 (45)	26 (68)	0.037	47 (78)	51 (85)	0.345
Scr, μmol/L	70.82 ± 14.52	72.87 ± 14.22	0.535	73.17 ± 15.38	75.55 ± 15.62	0.401
eGFR, mL/min/1.73 m^2^	111.69 ± 15.87	102.35 ± 12.73	0.006	106.51 ± 12.97	106.96 ± 14.06	0.855
TC, mmol/L	1.26 ± 0.38	1.19 ± 0.59	0.714	3.93 (3.51, 4.54)	3.96 (3.51, 4.36)	0.390
TG, mmol/L	4.05 ± 1.02	4.12 ± 0.84	0.537	0.96 (0.66, 1.50)	0.94 (0.74, 1.18)	0.354

ALT, alanine aminotransferase; Scr, serum creatinine; eGFR, estimated glomerular filtration rate; TC, total cholesterol; TG, triglyceride; NA, not available.

### 3.2 TMF effectiveness

#### 3.2.1 Reduction of qHBsAg and loss of HBeAg 

**FIGURE 2:**
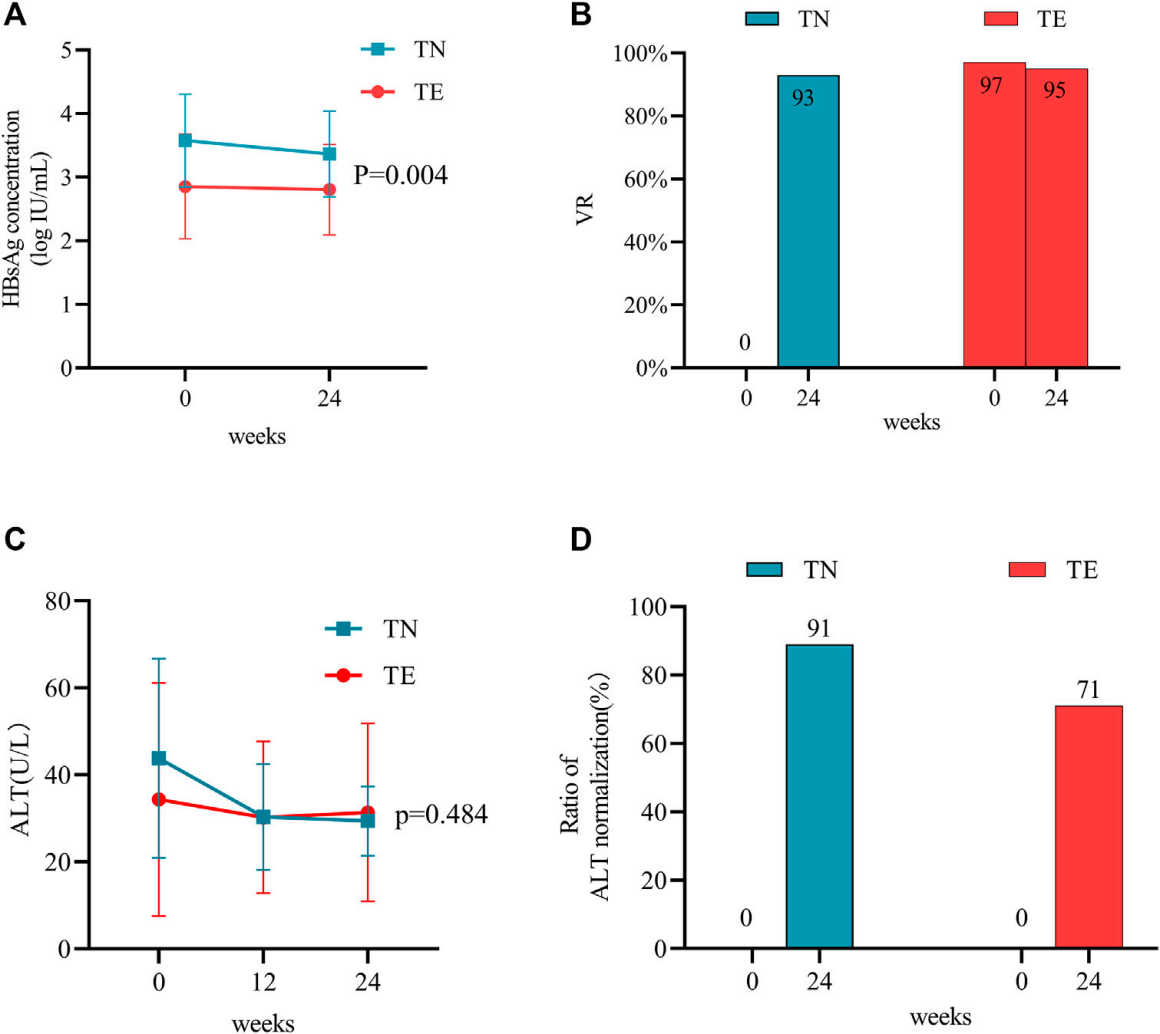
Effects of TMF on the antiviral efficacy between TN and TE groups. The effects of TMF on **(A)** HBsAg levels and **(B)** VR rates after 24 weeks of treatment. The effects of TMF on **(C)** ALT levels and **(D)** ratios of normal ALT after 12 and 24 weeks after treatment.

Serum qHBsAg levels were compared between the TN and TE groups (Figure 2A). In the TN group, the mean qHBsAg level decreased from 3.58 ± 0.73 log10 IU/mL to 3.36 ± 0.68 log10 IU/mL from baseline to week 24 (*p* = 0.000). Furthermore, the proportions of patients who were HBeAg-positive were 50% (27/54), 52% (28/54), and 46% (25/54) at baseline, week 12, and week 24, respectively. In the TE group, qHBsAg levels decreased from 2.85 ± 0.82 log10 IU/mL to 2.80 ± 0.71 log10 IU/mL during 24 weeks (*p* = 0.000). In addition, the proportions of patients who were HBeAg-positive were 41% (26/64), 39% (25/64), and 31% (20/64) at baseline, week 12, and week 24, respectively. qHBsAg levels were numerically higher in the TN group than those in the TE group throughout the period (*p* = 0.004).

#### 3.2.2 Virologic response

All patients in the TN group had detectable HBV DNA with a mean value of 5.16 ± 1.73 log10 IU/mL at baseline, and their VR rate was 93% (50/54) at week 24. There was a significant decrease in HBV DNA from baseline to week 24 (*p* = 0.001) (Figure 2B). Four TN patients did not achieve VR at week 24; their age was 28.25 ± 4.19 years; 75% (3/4) of them were HBeAg-positive and had relatively high levels of viremia (6.75 ± 1.41 log10 IU/mL). For patients in the TE group, VR rates were 97% (62/64) at baseline and 95% (61/64) at week 24 (Figure 2B). Of these, three patients in the TE group regained detectable HBV DNA during the 24 weeks of treatment (from undetectable HBV DNA to a mean level of 2.50 log10 IU/mL).

TN patients were further divided into two groups: HBeAg-positive and HBeAg-negative. Baseline data between the two groups are provided in Supplementary Table S1. Patients in the HBeAg-positive group had higher levels of serum HBsAg, HBV DNA, ALT, and AST (*p* < 0.05). After 24 weeks of follow-up, there was no statistical difference in VR rates, 88.9% (24/27) *vs.* 96.3% (26/27) (*p* = 0.603).

#### 3.2.3 The ratio of ALT normalization

For patients in the TN group, the mean ALT levels (U/L) were 43.83 ± 22.84, 30.33 ± 12.15, and 29.43 ± 7.96 at baseline, week 12, and week 24, respectively (Figure 2C). They were statistically decreased when compared to baseline measurements throughout the period (*p* = 0.000). The ratio of ALT normalization was 89% (25/28) at week 24 (Figure 2D).

For patients in the TE group, the mean ALT levels (U/L) were 34.37 ± 26.80, 30.27 ± 17.46, and 31.38 ± 20.44 at baseline, week 12, and week 24, respectively (Figure 2C). When compared with baseline measurements, no statistical differences in ALT levels were observed during 24 weeks (*p* = 0.456). Furthermore, the ratio of ALT normalization was 71% (10/14) at week 24 (Figure 2D).

Between the two groups, the ultimate ALT levels (*p* = 0.484) and ratios of normal ALT (*p* = 0.306) did not show statistical differences.

### 3.3 The safety profiles of TMF

#### 3.3.1 Renal dysfunction

**FIGURE 3:**
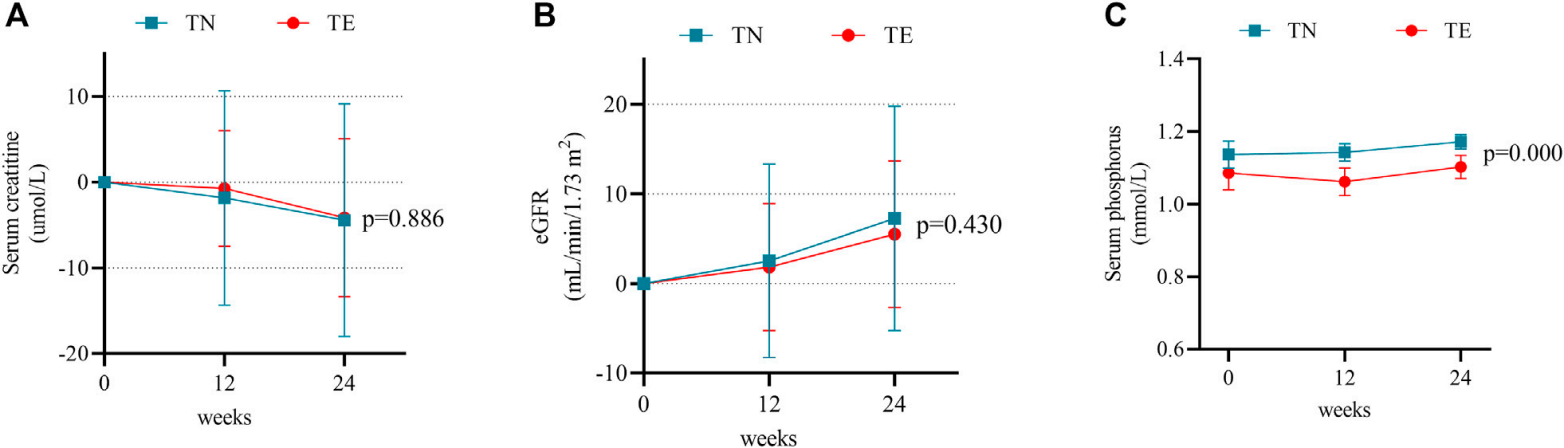
Changes in renal safety profiles. **(A)** Mean changes in serum creatinine at weeks 12 and 24 after treatment. Bars are expressed as mean ± SD. **(B)** Mean changes in the eGFR at weeks 12 and 24 after treatment. Bars are expressed as mean ± SD. **(C)** Serum phosphorus levels at weeks 12 and 24 after treatment. Bars are expressed as mean ± SD.

Mean changes in serum creatinine, eGFR, and serum phosphorus levels during the 24-week follow-up period were compared to baseline measurements in the TN and TE groups. In the TN group, serum creatinine decreased over time by a mean level of −1.83 ± 12.53 μmol/L at week 12 (*p* = 0.287) and −4.44 ± 13.55 μmol/L at week 24 (*p* = 0.019); eGFR increased at a level of 2.53 ± 10.79 ml/min/1.73 m^2^ at week 12 (*p* = 0.091) and 7.01 ± 12.49 ml/min/1.73 m^2^ at week 24 (*p* = 0.000); and serum phosphorus levels were 1.14 ± 0.14 mmol/L, 1.14 ± 0.09 mmol/L, and 1.17 ± 0.07 mmol/L at baseline, week 12, and week 24, respectively, and a statistical difference was shown between measurements at baseline and week 24 (*p* = 0.018) (Figures 3A–C).

In the TE group, serum creatinine decreased by a mean level of −0.73 ± 6.72 μmol/L at week 12 (*p* = 0.386) and −4.14 ± 9.33 μmol/L at week 24 (*p* = 0.001) (Figure 3A). Changes in eGFR were 1.83 ± 7.08 ml/min/1.73 m^2^ at week 12 (*p* = 0.043) and 5.50 ± 8.16 ml/min/1.73 m^2^ at week 24 (*p* = 0.000) (Figure 3B). Meanwhile, serum phosphorus levels were 1.08 ± 0.19 mmol/L, 1.06 ± 0.15 mmol/L, and 1.10 ± 0.13 mmol/L at baseline, week 12, and week 24, respectively, and the difference was not statistically significant between measurements at baseline and week 24 (*p* = 0.304) (Figure 3C).

Furthermore, there were no differences observed between the TN and TE cohorts in 24-week changes in serum creatinine and eGFR (*p* > 0.05). Serum phosphorus was numerically higher in the TN group and showed statistical differences when compared to that in the TE group at weeks 12 (*p* = 0.001) and 24 (*p* = 0.000).

#### 3.3.2 Blood lipids

**FIGURE 4:**
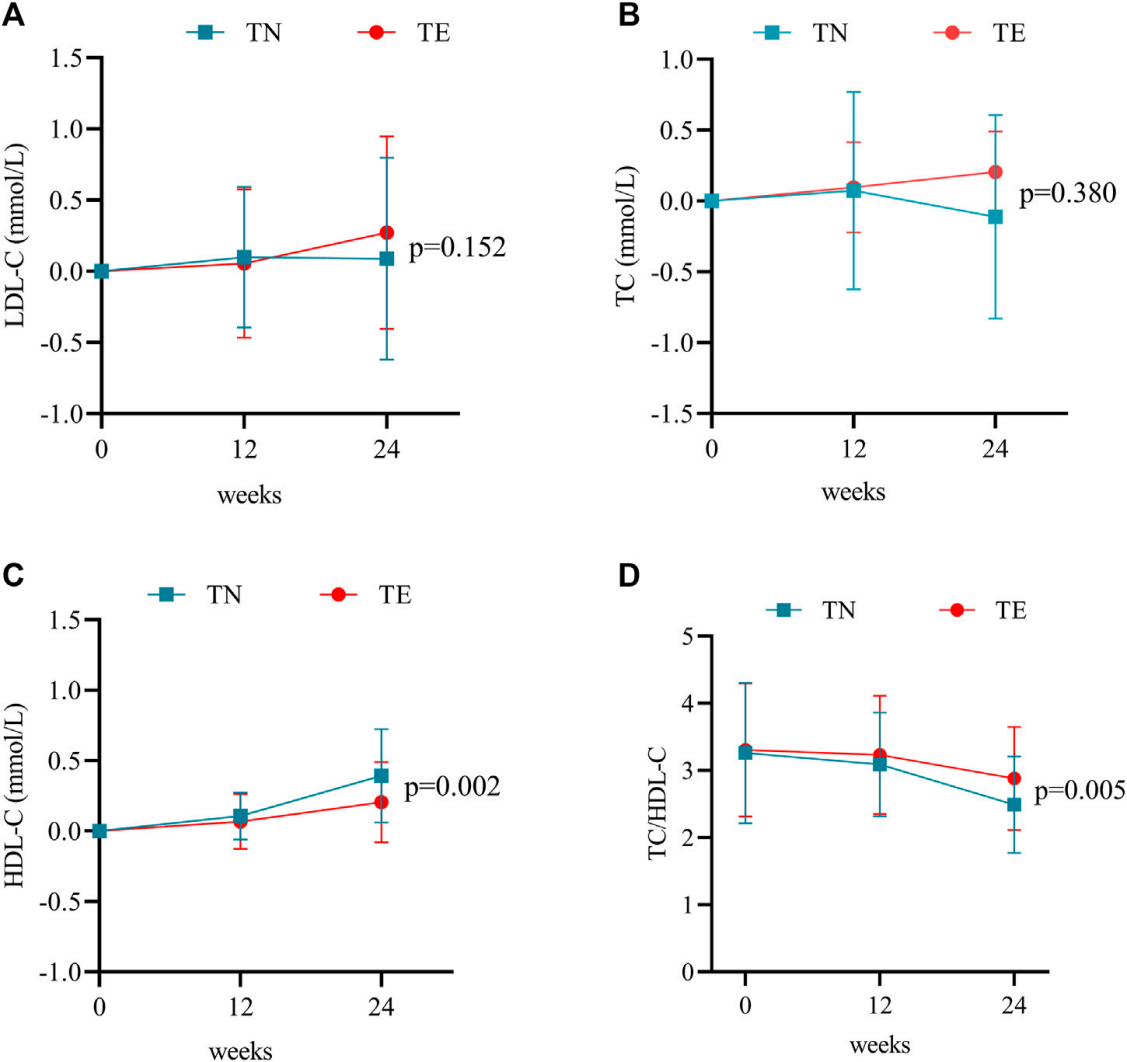
Changes in blood lipid profiles. **(A)** Mean changes in LDL-C at weeks 12 and 24 after treatment. Bars are expressed as mean ± SD. **(B)** Mean changes in TC at weeks 12 and 24 after treatment. Bars are expressed as mean ± SD. **(C)** Mean changes in HDL-C at weeks 12 and 24 after treatment. Bars are expressed as mean ± SD. **(D)** TC/HDL-C ratio at weeks 12 and 24 after treatment. Bars are expressed as mean ± SD.

In this study, blood lipids consisted mainly of TC, LDL-C, and HDL-C. In the TN group, mean changes in LDL-C levels were 0.10 ± 0.50 mmol/L at week 12 (*p* = 0.147) and 0.09 ± 0.71 mmol/L at week 24 (*p* = 0.360) (Figure 4A). TC increased by a mean of 0.07 ± 0.70 mmol/L at week 12 (*p* = 0.441) and −0.11 ± 0.72 mmol/L at week 24 (*p* = 0.257) (Figure 4B). Furthermore, changes in HDL-C levels were 0.10 ± 0.17 mmol/L at week 12 (*p* = 0.000) and 0.39 ± 0.33 at week 24 (*p* = 0.000) (Figure 4C). However, TC/HDL-C values decreased continuously from 3.26 ± 1.05 at baseline to 2.49 ± 0.72 at week 24 (*p* = 0.000) (Figure 4D).

For patients in the TE group, LDL-C increased by a mean of 0.06 ± 0.52 mmol/L at week 12 (*p* = 0.395) and 0.27 ± 0.68 mmol/L at week 24 (*p* = 0.002) (Figure 4A). Mean changes in TC levels were 0.09 ± 0.81 mmol/L at week 12 (*p* = 0.374) and 0.16 ± 0.99 mmol/L at week 24 (*p* = 0.895) (Figure 4B). Furthermore, changes in HDL-C levels were 0.07 ± 0.20 mmol/L at week 12 (*p* = 0.007) and 0.20 ± 0.29 mmol/L at week 24 (*p* = 0.000) (Figure 4C). However, TC/HDL-C values decreased continuously from 3.31 ± 0.99 at baseline to 2.88 ± 0.77 at week 24 (*p* = 0.001) (Figure 4D).

Statistical differences between the two groups were demonstrated by the changes in HDL-C levels (*p* = 0.002) and TC/HDL-C values (*p* = 0.005) during 24 weeks of treatment.

### 3.4 Effectiveness and safety of TMF *vs.* TAF

#### 3.4.1 TN patients in the PSM cohort

**FIGURE 5:**
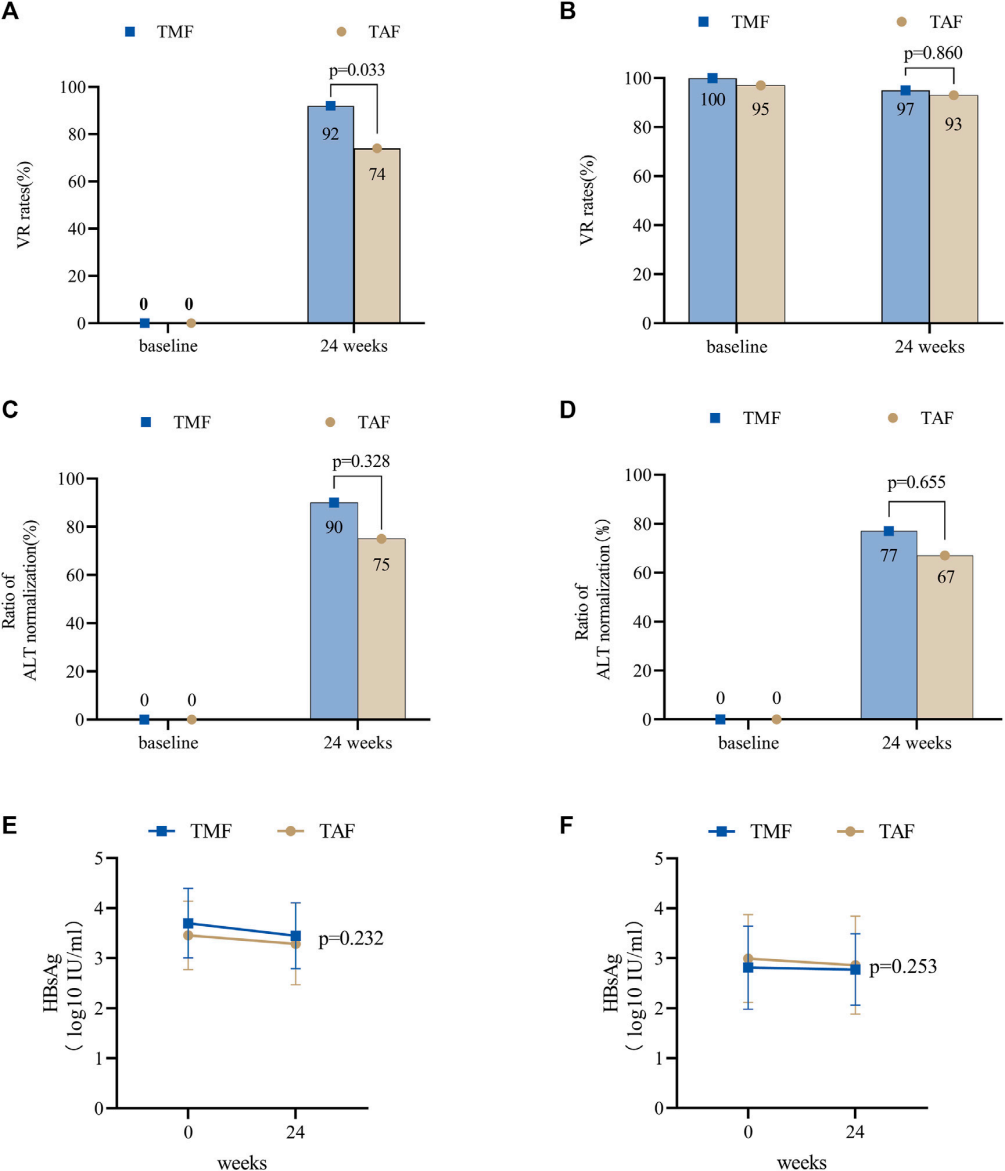
Antiviral efficacy of TMF and TAF after PSM in treatment-naive and treatment-experienced CHB patients. The effects of virologic response rates in treatment-naive patients **(A)** and treatment-experienced patients **(B)** after 24 weeks of treatment. The effects of the normal ALT ratios in treatment-naive patients **(C)** and treatment-experienced patients **(D)** after 12 and 24 weeks of treatment. The effects of HBsAg levels in treatment-naive patients **(E)** and treatment-experienced patients **(F)** after 24 weeks of treatment. Bars are expressed as mean ± SD.

In the TMF cohort, all patients had measurable HBV DNA at a level of 5.42 ± 1.72 log10 IU/mL, and the VR rate was 92% (35/38) at week 24 (Figure 5A). Furthermore, ALT levels (U/L) were 46.79 ± 22.97 at baseline, 29.50 ± 12.56 at week 12, and 29.08 ± 7.10 at week 24. Meanwhile, the ratios of normal ALT were 45% (17/38), 89% (34/38), and 92% (35/38) at baseline, week 12, and week 24, respectively. For patients whose baseline ALT was outside the normal range, the ALT normalization rate was 90% (19/21) at week 24 (Figure 5C). Furthermore, mean qHBsAg levels (log10 IU/mL) decreased from 3.70 ± 0.70 at baseline to 3.45 ± 0.66 at week 24 (Figure 5E).

In the TAF cohort, the baseline HBV DNA level was 4.57 ± 1.93 log10 IU/mL, and the VR rate was 74% (28/38) at week 24. Furthermore, ALT levels (U/L) were 39.34 ± 27.65 at baseline, 28.32 ± 12.30 at week 12, and 28.05 ± 12.69 at week 24 (Figure 5A). Meanwhile, the ratios of normal ALT were 68% (26/38), 84% (32/38), and 89% (34/38) at baseline, week 12, and week 24, respectively. The ALT normalization rate was 75% (9/12) at week 24 (Figure 5C). Furthermore, mean qHBsAg levels (log10 IU/mL) decreased from 3.46 ± 0.68 at baseline to 3.29 ± 0.82 at week 24 (Figure 5E).

At week 24, VR rates were numerically higher in the TMF cohort (*p* = 0.033), and no differences were found in ALT levels (*p* = 0.417), ratios of normal ALT (*p* = 1.000), and ALT normalization (*p* = 0.328).

**FIGURE 6:**
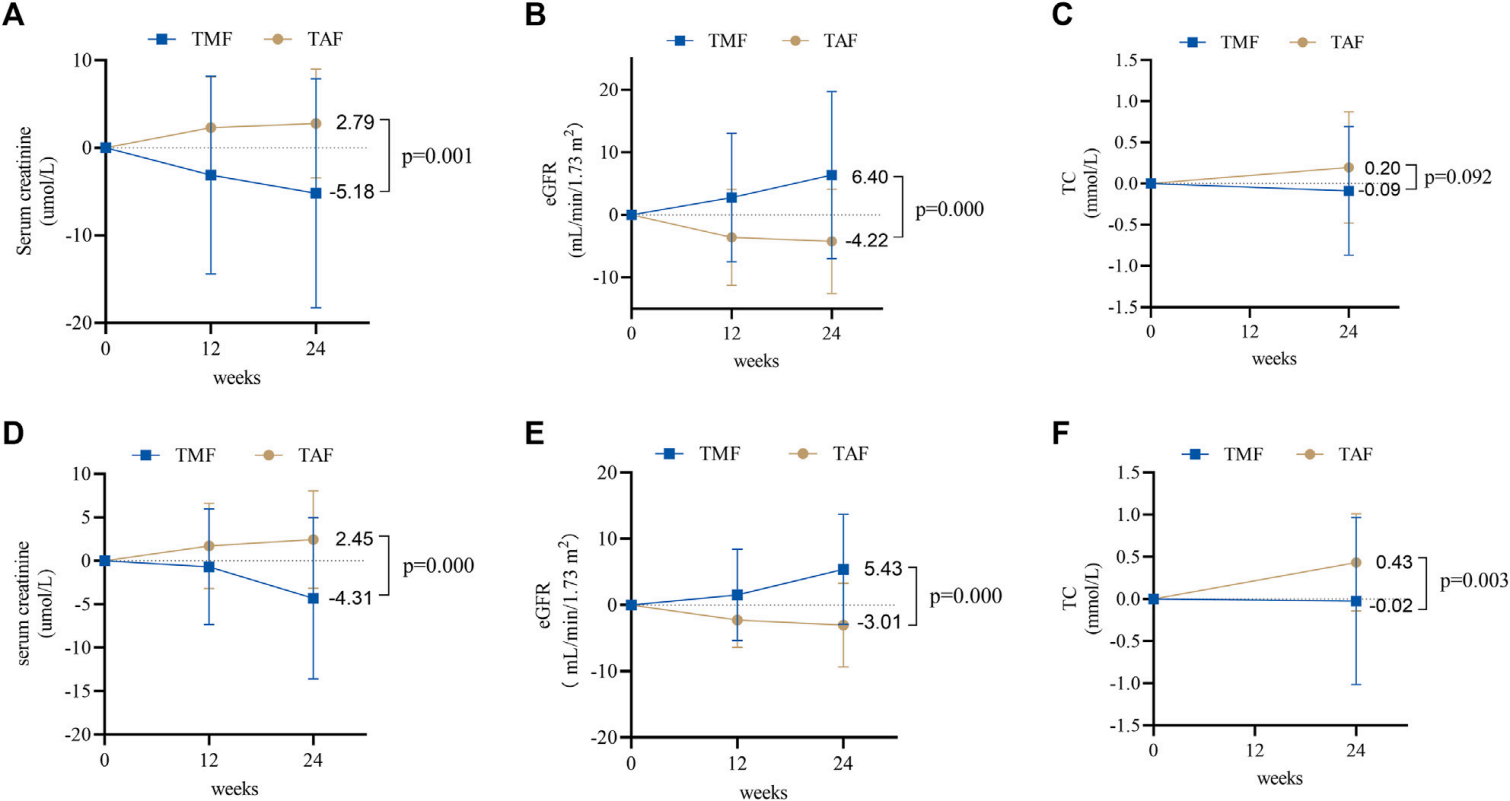
Changes in renal and blood lipid safety profiles between TMF and TAF cohorts. In treatment-naive patients, changes in serum creatinine **(A)** and eGFR **(B)** at weeks 12 and 24 after treatment; total cholesterol **(C)** at week 24 after treatment. In treatment-experienced patients, changes in serum creatinine **(D)** and eGFR **(E)** at weeks 12 and 24 after treatment; total cholesterol **(F)** at week 24 after treatment. All bars are expressed as mean ± SD.

The adverse effects of serum creatinine decreased in the TMF cohort and increased in the TAF cohort after 24 weeks (−5.18 ± 13.08 μmol/L *vs.* 2.79 ± 6.23 μmol/L, *p* = 0.001) (Figure 6A). Meanwhile, eGFR improved in the TMF cohort but worsened in the TAF cohort (6.40 ± 13.37 ml/min/1.73 m^2^ *vs.* −4.22 ± 8.34 ml/min/1.73 m^2^, *p* = 0.000) (Figure 6B). Changes in TC levels were −0.09 ± 0.78 mmol/L in the TMF cohort compared to 0.20 ± 0.67 mmol/L in the TAF cohort (*p* = 0.092) (Figure 6C).

#### 3.4.2 TE patients in the PSM cohort

In the TMF cohort, the VR rates were 100% (60/60) at baseline and 95% (57/60) at week 24 (Figure 5B). Meanwhile, ALT levels (U/L) were 34.29 ± 27.31, 28.23 ± 13.80, and 29.95 ± 19.05 at baseline, week 12, and week 24, respectively, and the ratios of normal ALT were 78% (47/60), 83% (50/60), and 90% (54/60) at baseline, week 12, and week 24, respectively. The ALT normalization rate was 77% (10/13) at week 24 (Figure 5D). Furthermore, qHBsAg decreased from 2.87 ± 0.70 log10 IU/mL at baseline to 2.78 ± 0.72 log10 IU/mL at week 24 (Figure 5F).

In the TAF cohort, the VR rates were 97% (58/60) at baseline and 93% (56/60) at week 24 (Figure 5B). Furthermore, ALT levels (U/L) were 29.01 ± 18.46, 24.87 ± 8.76, and 27.28 ± 14.62 at baseline, week 12, and week 24; meanwhile, the ratios of normal ALT at baseline, week 12, and week 24 were 85% (51/60), 92% (55/60), and 90% (54/60), respectively. Furthermore, the ALT normalization rate was 67% (6/9) at week 24 (Figure 5D). The mean levels of qHBsAg decreased from 2.99 ± 0.88 log10 IU/mL at baseline to 2.86 ± 0.98 log10 IU/mL at week 24 (Figure 5F).

There were no statistical differences between the two cohorts in VR rates (*p* = 0.860), ALT levels (*p* = 0.392), ratios of normal ALT (*p* = 0.318), or ALT normalization (*p* = 0.655) after 24 weeks of treatment.

Additionally, during 24-week treatment, serum creatinine decreased in the TMF cohort but increased in the TAF cohort [−4.31 ± 9.29 μmol/L *vs.* 2.45 ± 5.61 μmol/L, *p* = 0.000] (Figure 6D), and the eGFR increased in the TMF cohort but decreased in the TAF cohort [5.43 ± 8.30 ml/min/1.73 m^2^ *vs.* −3.01 ± 6.35 ml/min/1.73 m^2^, *p* = 0.000] (Figure 6E). In addition, TC levels after 24 weeks changed to −0.02 ± 0.99 mmol/L in the TMF cohort and 0.43 ± 0.58 mmol/L in the TAF cohort (*p* = 0.003) (Figure 6F).

## 4 Discussion

During 24 weeks of treatment in this retrospective, real-world study, the results demonstrated that TMF was highly effective in suppressing HBV replication in the blood. We found that nearly 93% of treatment-naive patients achieved a virologic response (HBV DNA <100 IU/mL) after 24 weeks of treatment. Furthermore, in treatment-experienced patients, TMF did not compromise antiviral effectiveness after switching from other antivirals. TMF also showed fewer side effects on renal function, as measured by decreased serum creatinine, increased eGFR, and increased serum phosphorus. In addition, TMF also showed a reduction in the ratio of TC to HDL-C levels, a predictor of cardiovascular disease risk. Furthermore, the comparison between TMF and TAF was also presented in this study. TMF was superior to TAF in suppressing HBV replication in treatment-naive patients. There were also opposite changing trends in the biomarkers of serum creatinine, eGFR, and total cholesterol.

The virologic response was an important endpoint during CHB antiviral treatment, which can improve liver inflammation and histology fibrosis and further improve clinical outcomes. One study with paired liver biopsies based on entecavir treatment demonstrated that high HBV DNA measurable rates after antiviral therapy were considered an independent risk factor for liver fibrosis progression (Sun et al., 2020). Furthermore, persisting detectable HBV DNA was of great importance in increasing the chances of hepatocellular carcinoma (Kim et al., 2017). In a recent phase III clinical trial, the VR rate (HBV DNA < 100 IU/mL) at week 48 was 82% in all patients (Liu et al., 2021), which was lower than that in the present study, where approximately 94% of patients had unmeasurable serum HBV DNA at week 24. The difference may result from lower baseline HBV DNA levels and a higher proportion of treatment-experienced patients, whose baseline HBV DNA was < 100 IU/mL in our study. More studies with less heterogeneity were needed to explore the exact ability of TMF to suppress viral replication.

When comparing treatment-naive patients who received TMF and TAF, virological response rates at week 24 were 92% in the TMF cohort and 74% in the TAF cohort, indicating that TMF can rapidly achieve undetectable serum HBV in treatment-naive patients. Furthermore, the result of the evaluation of the antiviral efficacy based on the HBeAg status revealed that the VR rate of the HBeAg-positive group is numerically lower, but the difference between the two groups indicated no statistical significance. A small sample may be the cause. However, this advantage was not shown in treatment-experienced patients. However, no matter which types of antiviral drugs were prescribed previously, the virologic response rates were maintained after switching to either TMF or TAF. Additionally, for treatment-experienced patients who had already achieved a virologic response, very few patients underwent HBV DNA re-detection during the treatment period. Only three patients in the TMF cohort and two patients in the TAF cohort had measurable HBV DNA again at week 24 (111, 119, and 2,450 IU/mL in the TMF cohort; 117 and 156 IU/mL in the TAF cohort). The common reasons for virologic breakthroughs were poor medication compliance, drug resistance, and so on (Hongthanakorn et al., 2011). As for the patients experiencing a virologic breakthrough in our cohort, they had the wrong medication or did not take the medication with a high-fat meal. Furthermore, some doses were occasionally missed. We speculated that the virologic breakthroughs in our study were associated with poor medication compliance.

Because of the difficulty of eliminating cccDNA in the hepatocyte, a functional cure was still the aim of CHB antiviral therapies. However, HBsAg seroclearance was a rare event. In untreated CHB patients, a spontaneous HBsAg loss occurred at a rate of approximately 1% per year, and HBeAg-negative patients were more likely to achieve HBsAg loss (Zhou et al., 2019). In a study of 5,409 CHB patients treated with entecavir or lamivudine, the results showed that the proportion was even lower (0.3% per year) (Kim et al., 2014). Additionally, a study in China demonstrated that the median rate of serum HBsAg reduction was 0.125 log10 IU/ml/year over 5 years (Seto et al., 2014) in CHB patients receiving ETV. The results of our study were comparable to those of the previous studies. Although the serum HBsAg levels showed a downward trend, their rate was too slow to achieve the goal of HBsAg seroclearance. Although the change in numerical numbers has statistical significance, it was less valuable from a clinical perspective. A total of 118 patients were treated with TMF, of which only 11 patients achieved HBeAg clearance, including three in the TN group and eight in the TE group. One patient in the TE group underwent HBsAg clearance with a baseline qHBsAg level of only 0.2 IU/mL. HBsAg clearance or seroconversion is difficult to achieve with TMF as is the case with other oral anti-HBV drugs.

As confirmed by previous studies, TAF was acknowledged to be superior in ALT normalization. The ratios in previous studies were approximately 50%–70% after 24 weeks of treatment (Chen et al., 2021; Pan et al., 2021; Lim et al., 2022). Our study showed that though the ratios of ALT normalization were numerically higher in the TMF cohort than those in patients who received TAF (90% *vs.* 75% of treatment-naive patients and 77% *vs.* 67% of treatment-experienced patients), no significant difference was observed between the two cohorts after 24 weeks of treatment. We could not conclude which treatment was better at normalizing ALT because of its short-term duration.

Renal tubule injuries are well-known adverse effects of tenofovir. TAF presented a better renal safety profile than TDF (Agarwal et al., 2018; Byun et al., 2022; Lim et al., 2022). As a new prodrug of tenofovir, renal dysfunctions of TMF were also demonstrated in phase III clinical study, manifesting as serum creatinine, which increased by 0.60 ± 8.988 μmol/L after 48 weeks of treatment. However, in our real-world study, we observed that TMF had no adverse effect on renal function. In this study, patients treated with TMF showed a decrease in serum creatinine and an increase in eGFR at baseline. TAF showed an opposite tendency when compared to TMF, indicating that TMF offers a better renal safety profile.

Serum lipids are risk factors associated with atherosclerotic cardiovascular disease (ASCVD), especially high LDL-C levels. However, LDL-C levels were not absolute indices for ASCVD risk prediction; almost half of all patients with coronary heart disease have normal LDL-C levels. Studies have proposed that the TC/HDL-C ratio may be another important risk predictor for ASCVD events in addition to LDL-C levels (Wen et al., 2019; Quispe et al., 2020). The results of the phase III clinical trial demonstrated that TMF represented an increase in the TC/HDL-C ratio (Liu et al., 2021). However, in our study, no matter how LDL-C levels changed, the TC/HDL-C ratio decreased continuously during the whole period. Furthermore, HDL-C levels were also increased in our study. The aforementioned biomarkers indicated that TMF showed fewer lipid disturbances and may be represented by fewer cardiovascular disease events in the future.

## 5 Strengths and limitations

This was the first study to highlight TMF and its comparison with TAF in the real world, demonstrating the potent antiviral effectiveness and better safety of TMF for the treatment of CHB. The currently published articles on TMF are results from phase III clinical trials. Compared with them, our research has broader inclusion and exclusion criteria for the CHB population, reducing the limitations of experimental conditions. Although the methods are not novel and the study design is similar to many articles, our research can fill the gaps in phase III clinical trials and improve our understanding of TMF. Furthermore, there are undeniably some other limitations. First, this is a single-center, retrospective study with a relatively small sample size and a short follow-up period. TMF is a novel prodrug of tenofovir that has been launched in China in June 2021. Due to the short marketing period, there are relatively few patients receiving TMF treatment, resulting in the small sample size of our study. Second, because of unavailable data on lipids in patients treated with TAF, our study did not analyze the differences in various blood lipids. Third, biomarkers, such as bone turnover markers, and dual-energy x-ray absorptiometry, which reflect bone abnormalities, were outside the scope of our study due to their high cost in the real world. We were also unable to acquire laboratory test data that assess renal proximal tubule damage because they are not commonly used in clinical practice, such as the urinary albumin-to-creatinine ratio, urinary retinol-binding protein-to-creatinine ratio, and urinary b2 microglobulin-to-creatinine ratio. Fourth, the sensitivity of the HBV DNA detection reagent used in this study is not high enough, making it difficult to identify CHB patients with hypoviremia, which may affect the research conclusion. For the reasons listed, long-term observational studies with large-sized samples and more comprehensive indicators are necessary.

## 6 Conclusions

In this 24-week study, TMF was shown to be highly effective in anti-HBV treatment with no obvious adverse effects on renal function and blood lipids. Although TMF revealed its superiority over TAF in serum HBV DNA in treatment-naive patients when, due to the small sample size and short follow-up time, further studies with larger cohorts and longer follow-up periods are needed to confirm our findings.

## Data Availability

The raw data supporting the conclusions of this article will be made available by the authors, without undue reservation.

## Supplementary material

SUPPLEMENTARY TABLE S1 Baseline clinical characteristics between the HBeAg-positive group and the HBeAg-negative group in TN patients.
