# Supplementary material for: Effectiveness and safety of tenofovir amibufenamide and its comparison with tenofovir alafenamide in patients with chronic hepatitis B: results from a retrospective real-world study
Source: Front Pharmacol. 2023 Jun 1;14:1165990. doi: 10.3389/fphar.2023.1165990 (PMC10267382; doi:10.3389/fphar.2023.1165990)
Supplement: Supplementary file 1 [file Table1.docx]

Supplementary Table 1. Baseline clinical characteristics between HBeAg-positive group and HBeAg-negative group among TN patients.

|  | HBeAg-  positive group (n=27) | HBeAg-  negative group  (n=27) | p value |
| --- | --- | --- | --- |
| Age(years) | 41.33±11.06 | 43.00±10.66 | 0.575 |
| Sex, n (%) |  |  |  |
| male | 14(52) | 15(56) | 0.785 |
| female | 13(48) | 12(44) |  |
| HBsAg, log10 IU/ml, | 3.90±0.39 | 3.47(2.95,3.76) | 0.001 |
| HBV DNA, log10 IU/ml, | 6.55(5.80,7.39) | 3.75(2.96,4.67) | 0.000 |
| PLT, x10^9^/L | 184(125,211) | 163(135,204) | 0.586 |
| TBI, mmol/L, | 14.6(10.8,19.3) | 18.7(11.7,20.6) | 0.177 |
| ALB, g/L | 42.76±3.27 | 45.13±5.62 | 0.064 |
| ALT, U/L | 48(38,76) | 27(19,53) | 0.002 |
| AST, U/L | 37(29,58) | 23(18,38) | 0.004 |
| Scr, umol/L | 71.30±15.67 | 69.00±15.50 | 0.591 |
| eGFR, mL/min/1.73m^2^ | 113.40±16.31 | 106.00±14.12 | 0.081 |
| serum phosphorous, mmol/L | 1.16±0.10 | 1.12±0.17 | 0.325 |
| TC, mmol/L | 3.82(3.44,4.17) | 4.09(3.84,4.27) | 0.139 |
| HDL-C, mmol/L | 1.25(1.17,1.33) | 1.32(1.19,1.55) | 0.139 |
| LDL-C, mmol/L | 3.21±0.96 | 3.09±0.91 | 0.626 |
| TC/HDL | 3.06(3.68,4.03) | 2.92(2.67,3.42) | 0.952 |

Abbreviations: PLT, platelet; TBIL, total bilirubin; ALB, albumin; AST, aspartate aminotransferase; ALT, alanine aminotransferase; Scr, serum creatine; eGFR, estimated glomerular filtration rate; TC, total cholesterol; HDL-C: high density liptein cholesterol; LDL-C, low density liptein cholesterol.
